# Using Expression Profiling to Understand the Effects of Chronic Cadmium Exposure on MCF-7 Breast Cancer Cells

**DOI:** 10.1371/journal.pone.0084646

**Published:** 2013-12-20

**Authors:** Zelmina Lubovac-Pilav, Daniel M. Borràs, Esmeralda Ponce, Maggie C. Louie

**Affiliations:** 1 Systems Biology Research Centre – Bioinformatics, School of Bioscience, University of Skövde, Skövde, Sweden; 2 Department of Natural Sciences and Mathematics, Dominican University of California, San Rafael, California, United States of America; 3 College of Pharmacy, Touro University of California, Vallejo, California, United States of America; University of California Davis, United States of America

## Abstract

Cadmium is a metalloestrogen known to activate the estrogen receptor and promote breast cancer cell growth. Previous studies have implicated cadmium in the development of more malignant tumors; however the molecular mechanisms behind this cadmium-induced malignancy remain elusive. Using clonal cell lines derived from exposing breast cancer cells to cadmium for over 6 months (MCF-7-Cd4, -Cd6, -Cd7, -Cd8 and -Cd12), this study aims to identify gene expression signatures associated with chronic cadmium exposure. Our results demonstrate that prolonged cadmium exposure does not merely result in the deregulation of genes but actually leads to a distinctive expression profile. The genes deregulated in cadmium-exposed cells are involved in multiple biological processes (i.e. cell growth, apoptosis, etc.) and molecular functions (i.e. cadmium/metal ion binding, transcription factor activity, etc.). Hierarchical clustering demonstrates that the five clonal cadmium cell lines share a common gene expression signature of breast cancer associated genes, clearly differentiating control cells from cadmium exposed cells. The results presented in this study offer insights into the cellular and molecular impacts of cadmium on breast cancer and emphasize the importance of studying chronic cadmium exposure as one possible mechanism of promoting breast cancer progression.

## Introduction

The International Agency for Research on Cancer and the National Toxicology Program of the United States of America have classified cadmium as a category I human carcinogen [1,2] because increasing evidence shows both occupational and non-occupational exposure to cadmium are associated with cancers of the lung, prostate, pancreas, kidney, liver, stomach, and urinary bladder [3-9]. Cadmium exposure has also been correlated with an increase in the incidence of breast cancer [10-12]. Recent studies have identified significantly higher levels of cadmium in tumor tissues as well as in other biological samples from tumor patients [8,13,14]. Specifically, Strumylaite et al. found that patients with malignant breast cancer had accumulated significantly higher levels of cadmium when compared to patients with benign tumors [13] offering further support for a possible relationship between cadmium and breast cancer progression.

Several studies have shown that cadmium has the ability to mimic the biological functions of estrogen in breast cancer cells by activating the estrogen receptor [11,15-20]. Previous work from our lab and others has suggested that cadmium can promote MCF-7 breast cancer cell growth via ERα [11,16,19]. Animal studies have revealed that, similar to estrogen, cadmium promotes neoplastic growth, increases uterine weight, induces changes in the uterine lining, and increases the density of mammary glands in rats and mice [17,21-23]. Collectively, these studies demonstrate that cadmium functions as a hormone-disruptor and metalloestrogen and is an important contributor to the development of breast cancer.

Several studies have also shown that cadmium exposure is associated with the development of more malignant tumors [6,24,25]. Furthermore, results from our recent study have suggested that cells chronically exposed to cadmium acquire a more metastatic phenotype that includes an increased ability to migrate and invade [20]. We demonstrated that prolong exposure to cadmium induces cellular changes brought about by changes in the expression of genes such as SDF-1; however a comprehensive molecular understanding of how chronic exposure to cadmium mediates these changes in phenotype remains unclear. 

In this study, microarray analysis was used to investigate the effect of prolonged cadmium exposure on gene expression by comparing the global gene expression of MCF-7 breast cancer cells chronically exposed to cadmium with that of control MCF-7 cells. Genes that were differentially expressed in cadmium-adapted cells were further analyzed. 

## Materials and Methods

### Cell Culture

MCF-7 cells were obtained from American Type Culture Collection (ATCC, Manassas, VA). MCF-7 cells were maintained in complete media: Dulbecco’s Modified Eagle Medium (DMEM) plus 10% fetal bovine serum and 1% penicillin and streptomycin (Hyclone, Logan, UT). Cadmium-adapted cell lines (MCF-7-Cd4, -Cd6, -Cd7, -Cd8 and -Cd12) were derived as previously described [20], and maintained in complete media but supplemented with 10^-7^ M CdCl_2_.

### Affymetrix Microarray

Equal numbers of MCF-7 and MCF-7-Cd4, -Cd6, -Cd7, -Cd8, and -Cd12 cells were plated in 10 cm plates and semi-synchronized by serum starvation. Twenty-four hours later, media containing hormone (DMEM+ 10% FBS) were added back to cells. Cells were harvested 10 hours later for total RNA isolation using Direct-zol™ RNA MiniPrep (Zymo Research Corporaton, Irvine, CA) according to manufacturer’s protocol. In short, cells were lysed with TRI-Reagent and mixed with equal volume of 100% ethanol. Mixture was transferred to a Direct-zol column and following several washes, total RNA was eluted using nuclease-free water. Microarray experiments were performed by the QB3-Functional Genomics Lab at UC Berkeley (Berkeley, CA) using GeneChip^®^ Human Gene 1.0 ST arrays (Affymetrix, Santa Clara, CA). Expression signals were extracted and normalized by means of the Expression Console software (Affymetrix) applying the Robust Multichip Average (RMA) normalization method [26]. The complete microarray expression data are available at the NCBI´s Gene Expression Omnibus (GEO) (accession GSE52404).

### Quantitative Reverse Transcriptase-Polymerase Chain Reaction (qRT-PCR)

Total RNA was isolated from cells using Direct-zol RNA MiniPrep kit according to manufacturer’s protocol (Zymo Research Corporation, Irvine, CA). Three micrograms (μg) of total RNA were used for the reverse transcription reaction with oligo-dT_18_ primers and Moloney Murine Leukemia Reverse Transcriptase (M-MLV RT, Promega, Madison,WI). Gene expression was monitored using SYBR-green based qRT-PCR (Viia-7; Applied Biosystems, Life Technologies, Grand Island, NY). All primers were synthesized by Integrated DNA Technologies, Inc. (IDT; San Diego, CA). The primer sequences are listed in Table 1. 

**Table 1:** Primer Sequences.

**MT1X_F_** :CTCTTGATCGGGAACTC	**MT1X_R_**: GGTTGCTCTATTTACATCTG
**ANXA3**_F_ :ATGCCCAGATTCTCTATAA	**ANAX3_R_**: GATCTGGACACCATTATTC
**MT2A _F_**:AACCTGTCCCGACTCTA	**MT2A_R_**:GGAAGTCGCGTTCTTTAC
**CCNE2 _F_**:GGGAGTACCAAAGAAATTAT	**CCNE2_R_**:AGTTTAGGTCAAGTGTTAAG
**CCNB1_F_**: GTACCCTCCAGAAATTGGTGA	**CCNB1_R_**: GAC TAC ATT CTT AGC CAG GTG
**BUB1 _F_**: GGCTCCTACACTTCCTGATATTT	**BUB1_R_**: CTGGCTCCTGTGGGTTTATT
**CENPF _F_**: GTTCAAGAAGCTGGAGATAG	**CENPF_R_**:CCCTCGGGAATCTTGTTTAG

### Hierarchical clustering

Hierarchical clustering of the samples was performed to determine if cadmium exposed samples could be separated from the control samples. Clustering was performed with Java-based desktop application MultiExperiment Viewer (MeV) [27] by using the average linkage rule and Pearson correlation as the similarity metric. 

### Identifying differentially expressed (DE) genes

To understand the effects of chronic cadmium exposure on gene expression in breast cancer, two control MCF-7 parental cell lines and five different clonal cadmium-adapted cell lines (MCF-7-Cd4, -Cd6, -Cd7, -Cd8, and -Cd12)— previously derived from cells chronically exposed to cadmium— were used for microarray analysis. Expression signals were extracted and normalized by means of the Expression Console software (Affymetrix) applying the Robust Multichip Average (RMA) normalization method [26]. The data were background corrected, normalized (Quantile) and summarized (MedianPolish). Differentially expressed genes were identified with Significance Analysis of Microarrays – SAM [27,28]. SAM relies on a modified *t*-test to determine the significance of the expression level of each gene. SAM then uses permutations to estimate the false discovery rate (FDR) [29] i.e. the fraction of false positive genes. Cut-off for FDR was set to 0.05 in order to identify genes with significantly aberrant expression levels to be further analyzed. 

### Functional enrichment of the DE genes

A total of 795 DE genes were used as input for further analysis. A singular enrichment analysis (SEA) was applied to identify the annotation terms that are overrepresented in our gene list. Following SEA, two additional enrichment analysis tools were used; the first was Database for Annotation, Visualization and Integrated Discovery (DAVID; http://david.abcc.ncifcrf.gov/) [30], which relies on a modified Fisher's exact test (EASE score), and the second is Onto-Express [31,32] (http://vortex.cs.wayne.edu/projects.htm) which implements a hypergeometric statistical method. Both of the tools rely on Gene Ontology (GO) as a primary annotation source, but also integrate other annotation sources. 

Furthermore, genes that are known to be associated with breast cancer were identified using Genes-to-Systems Breast Cancer Database - G2SBC (http://www.itb.cnr.it/breastcancer/) [33], which gathers information about genes that have been previously reported in literature to be associated with breast cancer. Gene Expression Atlas - GXA (http://www.ebi.ac.uk/gxa) from EMBL-EBI database [34] was also used for further investigation of DE genes by searching for previously described gene expression patterns associated with different biological/experimental conditions, such as estrogen expression. 

## Results

### Hierarchical clustering

**Figure 1:**
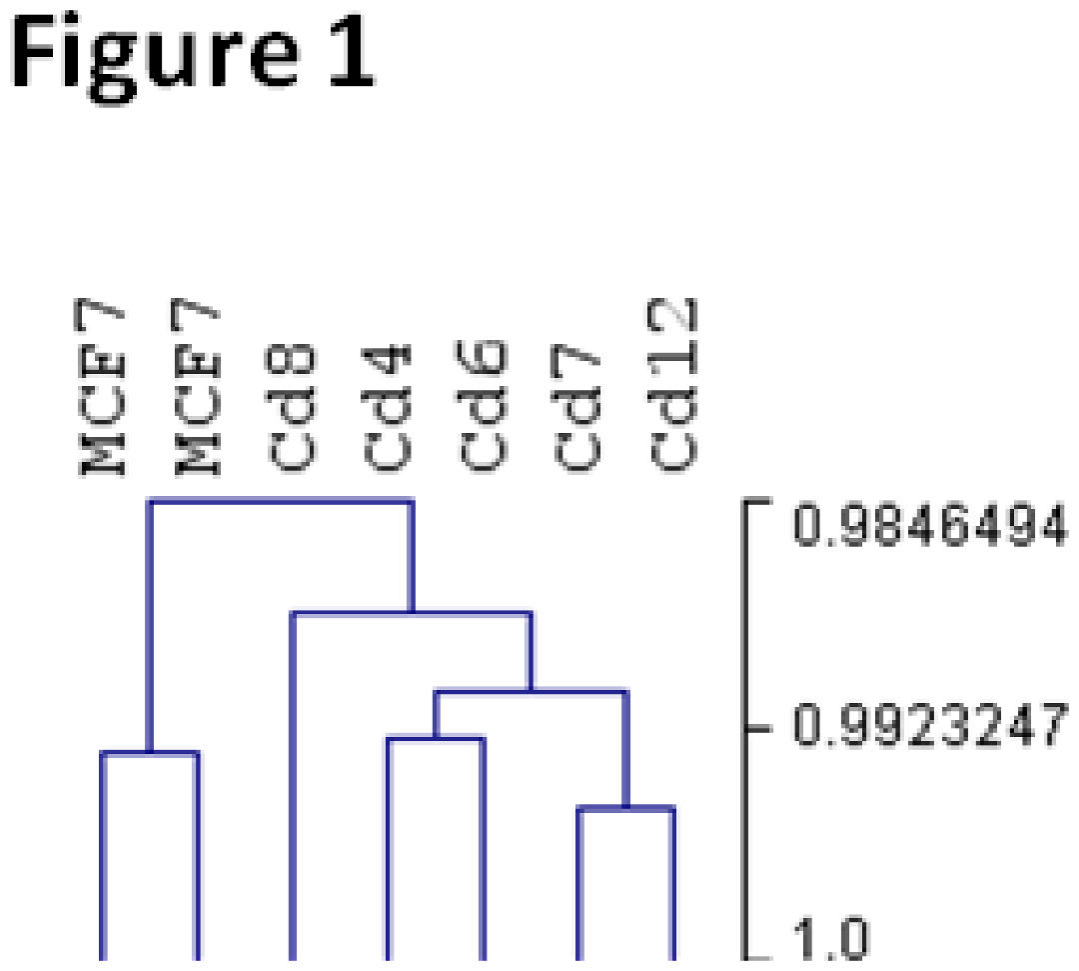
Dendrogram of hierarchical clustering showing a clear distinction between control MCF-7 and cells chronically exposed to cadmium.

We performed hierarchical clustering of the samples to see if there is a separation between control MCF-7 cells and those treated with cadmium for over 6 months. Figure 1 shows a dendogram with two major branches, confirming the discrimination between control and cadmium-treated samples. This suggests that genes differentially expressed in breast cancer cells chronically exposed to cadmium can be identified using this set of microarray data.

### Identifying differentially expressed genes

SAM analysis was used to identify differentially expressed (DE) genes, and this revealed 944 DE genes at FDR<0.05. Among these are 371 over-expressed and 573 are under-expressed genes. The gene list was then filtered by removing duplicate gene symbols resulting in 795 DE genes (Table S1), which were used for further analysis. Table 2 shows a list of the top 30 over-expressed genes, along with the associated *q*-value (measure of significance in terms of false discovery rate), fold change log (FClog) and gene description. A corresponding list of 30 under-expressed genes can be found in Table 3. For a complete list of DE genes, see supplemental information (Table S1).

**Table 2:** 30 over-expressed genes with lowest *q*-value.

**Gene symbol**	**FClog**	***q*-value**	**Gene description**
*ANXA3*	2.15	0.00E+00	annexin A3
ULBP2	1.65	1.67E-02	UL16 binding protein 2
MT1F	1.46	1.67E-02	metallothionein 1F
SCARNA9	1.23	1.67E-02	small Cajal body-specific RNA 9
MT1L	1.21	1.42E-02	metallothionein 1L
*DKK1*	1.18	1.42E-02	dickkopf homolog 1
*PDLIM1*	0.98	1.51E-02	PDZ and LIM domain 1
PTP4A3	0.98	1.67E-02	protein tyrosine phosphatase type IVA, member 3
ANXA2P2	0.97	1.42E-02	annexin A2 pseudogene 2
MT1X	0.97	5.87E-03	metallothionein 1X
PRSS8	0.90	6.81E-03	protease, serine, 8
CERCAM	0.85	1.42E-02	cerebral endothelial cell adhesion molecule
*CRABP1*	0.83	1.67E-02	cellular retinoic acid binding protein 1
CBFA2T3	0.79	1.51E-02	core-binding factor, runt domain, alpha subunit 2; transloc to 3
DECR1	0.76	1.64E-02	2,4-dienoyl CoA reductase 1
FSTL3	0.75	1.19E-02	follistatin-like 3
*UCP2*	0.73	1.64E-02	uncoupling protein 2
FKBP9	0.73	1.62E-02	FK506 binding protein 9
ZNF286B	0.71	1.67E-02	zinc finger protein 286B
*MT2A*	0.66	0.00E+00	metallothionein 2A
TRADD	0.62	1.19E-02	TNFRSF1A-associated via death domain
CRYL1	0.62	1.42E-02	crystallin, lambda 1
ZNHIT2	0.59	1.67E-02	zinc finger, HIT-type containing 2
ATP2A3	0.54	1.67E-02	ATPase, Ca++ transporting, ubiquitous
*SRD5A1*	0.53	1.64E-02	steroid-5-alpha-reductase, alpha polypeptide 1
RAB32	0.52	1.67E-02	RAB32, member RAS oncogene family
TMEM8A	0.49	1.58E-02	transmembrane protein 8A
ZP1	0.49	1.19E-02	zona pellucida glycoprotein 1 (sperm receptor)
SLC15A4	0.45	1.19E-02	solute carrier family 15, member 4
*CCNE1*	0.45	1.51E-02	cyclin E1

Underlined genes are known to be associated with breast cancer.

**Table 3:** 30 under-expressed genes with lowest *q*-value.

**Gene symbol**	**FClog**	***q*-value**	**Gene description**
SH3GL3	-0.52	5.16E-03	SH3-domain GRB2-like 3
NPSR1	-0.62	0.00E+00	neuropeptide S receptor 1
*TK1*	-0.62	6.47E-03	thymidine kinase 1, soluble
RHEBL1	-0.65	0.00E+00	Ras homolog enriched in brain like 1
GPR128	-0.68	5.54E-03	G protein-coupled receptor 128
REXO1L2P	-0.73	5.62E-03	REX1, RNA exonuclease 1 homolog (S. cerevisiae)-like 2
LPAR6	-0.75	5.83E-03	lysophosphatidic acid receptor 6
ABCA6	-0.77	0.00E+00	ATP-binding cassette, sub-family A
SPDYE1	-0.82	0.00E+00	speedy homolog E1 (Xenopus laevis)
*PGK1*	-0.83	0.00E+00	phosphoglycerate kinase 1
TAF9B	-0.87	0.00E+00	RNA polymerase II, TATA box binding protein-associated factor
SPDYE8P	-0.91	0.00E+00	speedy homolog E8 (Xenopus laevis)
SNORD115-20	-0.92	5.79E-03	small nucleolar RNA, C/D box 115-11
SSX2	-0.95	0.00E+00	synovial sarcoma, X breakpoint 2
OR2AT4	-1.05	5.87E-03	olfactory receptor, family 2, subfamily AT, member 4
SSX4	-1.08	0.00E+00	synovial sarcoma, X breakpoint 4
OCM2	-1.13	6.06E-03	oncomodulin 2
TBX4	-1.17	0.00E+00	T-box 4
OR7E87P	-1.18	0.00E+00	olfactory receptor, family 7, subfamily E, member 87
LOC652493	-1.32	5.87E-03	similar to pre-B lymphocyte gene 1 gene:ENSG00000241755
OCM	-1.33	0.00E+00	oncomodulin
CYP24A1	-1.38	0.00E+00	cytochrome P450, family 24, subfamily A, polypeptide 1
SNORD59B	-1.55	5.87E-03	small nucleolar RNA, C/D box 59B
IGLJ3	-1.63	0.00E+00	mRNA for scFv collagenase IV antibody
IGHA1	-1.64	0.00E+00	cDNA FLJ46621 fis, clone TLUNG2001445
SUMO1P1	-1.88	0.00E+00	SUMO1 pseudogene 1
DUB3	-1.88	0.00E+00	ubiquitin specific peptidase 17-like 2
DUB4	-2.00	0.00E+00	ubiquitin specific peptidase 17-like 6 (pseudogene)
USP17	-2.07	0.00E+00	ubiquitin specific peptidase 17
IGHD	-2.11	0.00E+00	immunoglobulin heavy constant delta

Underlined genes are known to be associated with breast cancer.

Amongst the over-expressed genes in Table 2, some genes are expected to show differential gene expression after cadmium exposure, including those associated with endocrine disruption and heavy metal toxicity. Prior to identifying these genes, an initial search of all listed genes was done using Gene Expression Atlas (GXA) to find genes with expression patterns associated with estrogenic effects and metal/cadmium toxicity. In the case of endocrine disruption effects, the genes that were up-regulated and associated with this function include ULBP2, MT1F, DKK1, PDLIM1, PTP4A3, ANXA2P2, MT1X, PRSS8, CERCAM, CRABP1, CBFA2T3, DECR1, FSTL3, UCP2, FKBP9, ZNF286B, MT2A, TRADD, CRYL1, ZNHIT2, ATP2A3, SRD5A1, RAB32, ZP1, SLC15A4, CCNE1 and ANXA3 (Table 2), while genes SH3GL3, TK1, CYP24A1, PGK1, TAF9B, SSX2, TBX4, SPDYE1, RHEBL1 and ABCA6 were down-regulated (Table 3). We also identified DE genes associated with heavy metal toxicity and these genes include MTJF, MT1L, MT1X, and MT2A which are up-regulated (Table 2), while CYP24A1 and TK1 genes associated with calcium homeostasis and zinc binding, respectively are down-regulated (Table 3). 

**Figure 2:**
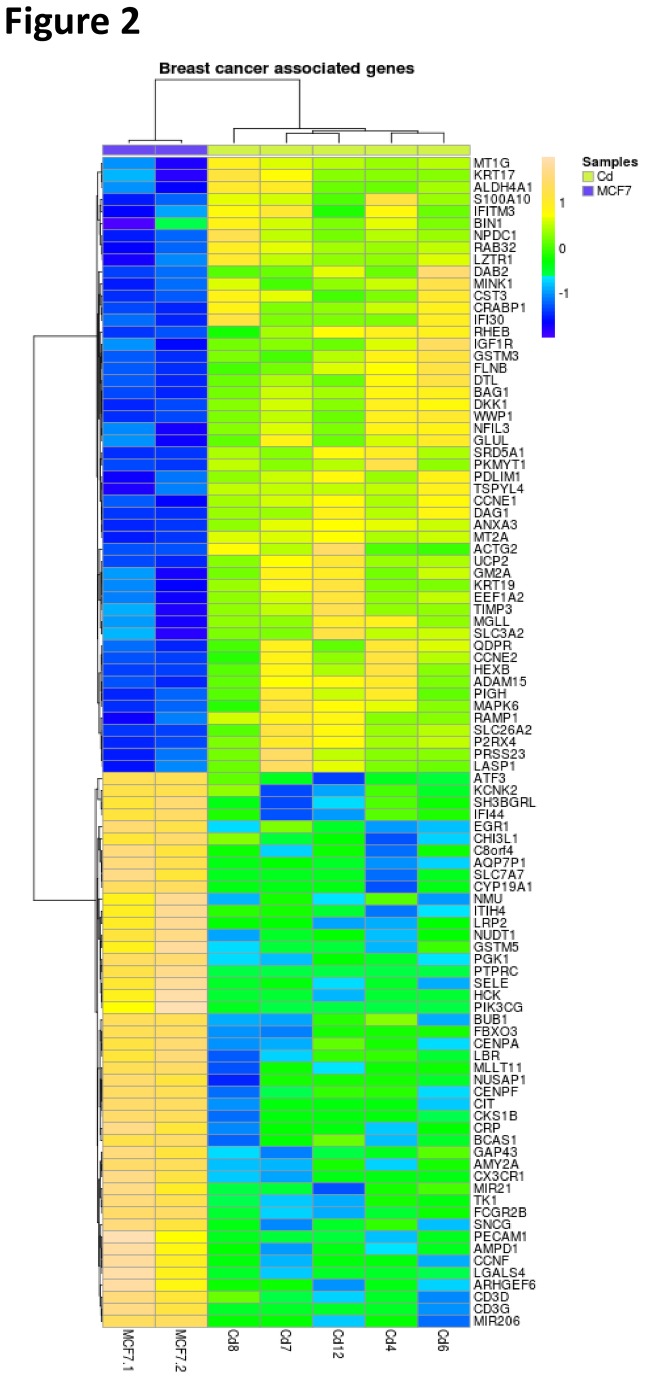
Heat map showing expression of 97 breast cancer associated genes that are differentially expressed between control and cadmium-adapted cells. Each row represents a gene and each column represents a sample.

Apart from this, several of the DE genes were already known to be associated with breast cancer, not unexpected since cadmium has been shown to be involved in the development and progression of breast cancer [17,19,20,35,36]. Among the genes in Table 2, eight are known to be associated with breast cancer including: ANXA3 (Annexin A3), an inhibitor of phospholipase A2; DKK1 (Dickkopf), a precursor protein that inhibits the Wnt signaling pathway; PDLIM1 (DZ and LIM domain protein 1), a protein that has been shown to interact with ERα; CRABP1 (cellular retinoic acid-binding protein), a protein that may play a role in retinoic acid-mediated differentiation and proliferation; SRD5A1 (5-a reductase type 1), an enzyme involved in the metabolism of progesterone; MT2A (metallothionein 2A), metal response protein; UCP2 (uncoupling protein 2), an inner mitochondrial membrane anion carrier that negatively regulates reactive oxygen species production; and CCNE1 (cyclin E1), a G1/S cyclin that regulates cell cycle progression. Two additional genes in Table 3— TKI (Thymidine kinase 1) and PGK1 (Phosphoglycerate kinase 1)— are also known to be associated with breast cancer. Other than the genes highlighted in Tables 2 and 3, analysis of the complete list of DE genes identified an additional 87 genes with known breast cancer association. These are all summarized in the heat map shown in Figure 2. Specifically, the heat map is used to highlight the expression pattern of breast cancer associated genes in the cadmium samples in comparison to the control samples with the dendrogram of the genes shown on the left-hand side, and the dendrogram on top depicting the different samples. Hierarchical clustering method based on Pearson dissimilarity metric was used to create the dendrograms. The heat map also indicates that the set of breast cancer associated genes follows similar expression pattern as the whole set of DE genes, clearly separating control cells from cadmium-exposed cells. This subset of genes is differentially expressed in all five clonal cadmium cell lines suggesting that a common gene expression signature does exist in cells chronically exposed to cadmium.

### Functional enrichment analysis of the DE genes

**Figure 3:**
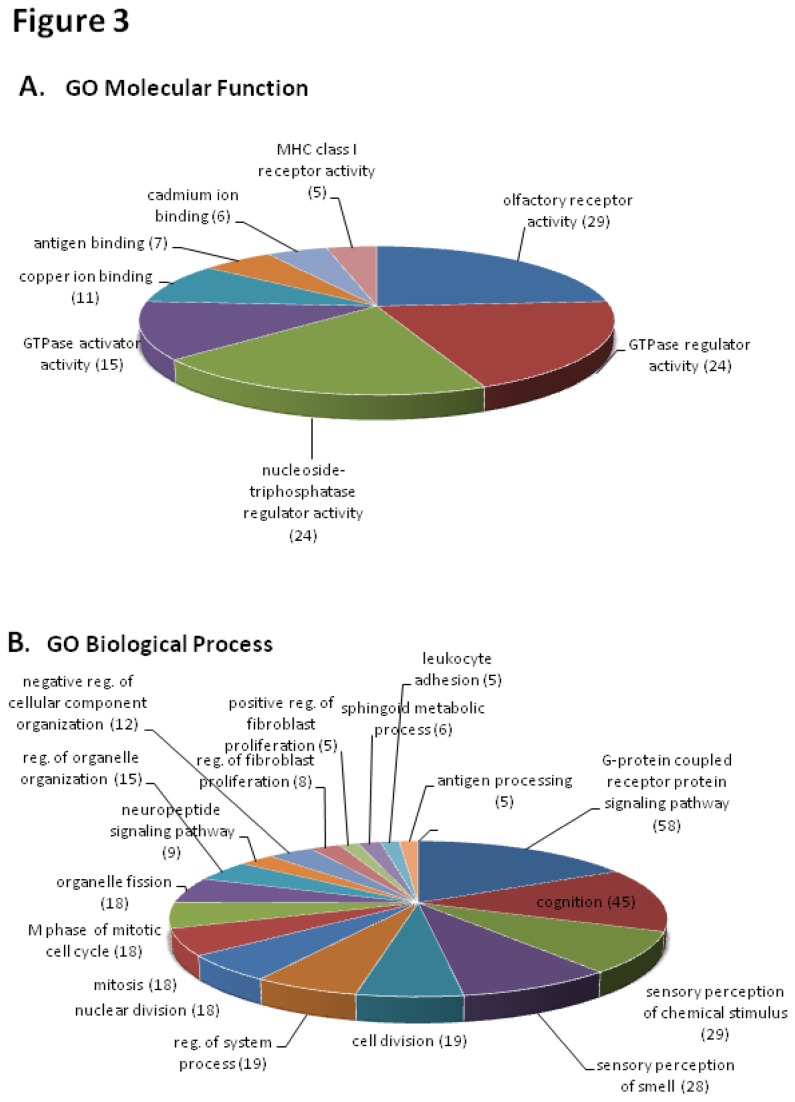
Over-represented Gene Ontology (GO) annotation among differentially expressed genes. GO categories identified using Database for Annotation, Visualization and Integrated Discovery (DAVID; http://david.abcc.ncifcrf.gov/). **A**) Biological Process and **B**) Molecular Function.

We performed gene annotation enrichment analysis of DE genes, focusing on Gene Ontology (GO), which is the *de facto* method for functional annotation and also used as a primary annotation source in the majority of enrichment tools. Initially, we applied DAVID to identify significant annotation terms GO molecular function (GO MF) and GO biological process (GO BP) terms— with the purpose of finding important biological functions and processes that characterize the impact of the DE genes identified in our study. Out of 795 DE genes, there were 783 hits found in DAVID. We applied a cut-off *p*<0.05 to derive significantly over-represented GO MF and GO BP terms (Figure 3A and B). Among the GO BP terms identified by DAVID, the most significantly enriched term is “regulation of fibroblast proliferation” (*p*-value = 2.29E-04), with 8 genes assigned to it. One of the up-regulated genes in this set is IFI30, interferon gamma-inducible protein 30, which is linked to breast cancer [37,38]. Other GO BP terms of interest include “cell division” (19 genes), “nuclear division” (18 genes), and “G-protein coupled receptor protein signaling pathway” (58 genes). Many of the genes assigned with these terms are known to contribute to cancer cell growth and development. Among the most significantly enriched GO MF terms are the “cadmium ion binding” (*p* = 1.6E-05) and “copper ion binding” (*p* = 2.5E-04) (Figure 3A). 

**Figure 4:**
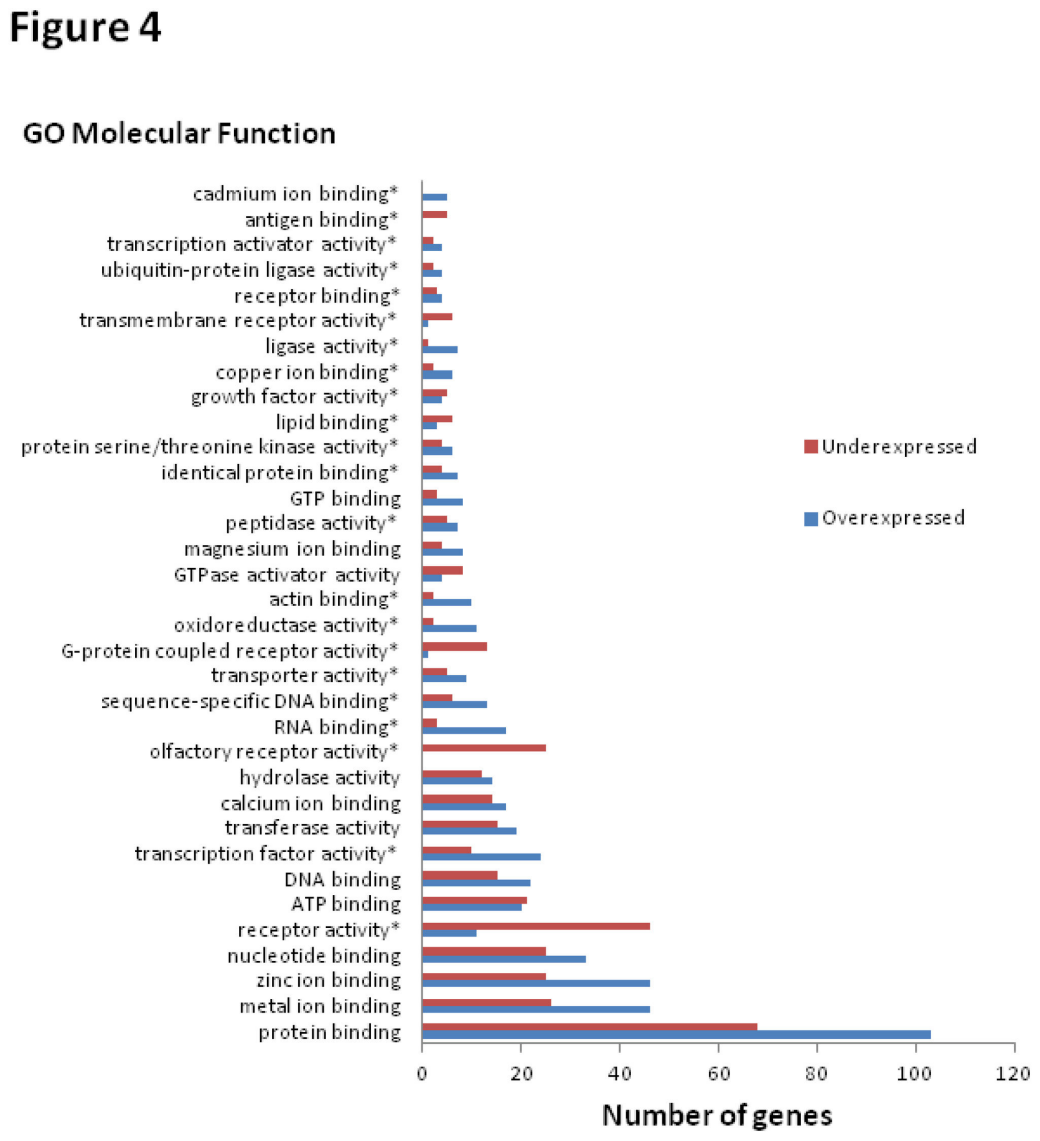
Gene Ontology (GO) categories denoting Molecular Function (MF) information for differentially expressed genes. Information is derived using Onto-Express (http://vortex.cs.wayne.edu/projects.htm). Diagram shows the number of under- and over-expressed genes for each GO MF. The MF marked with stars denotes a statistically significant group (*p*<0.05). Only the groups with a gene number ≥ 5 are shown.

**Figure 5:**
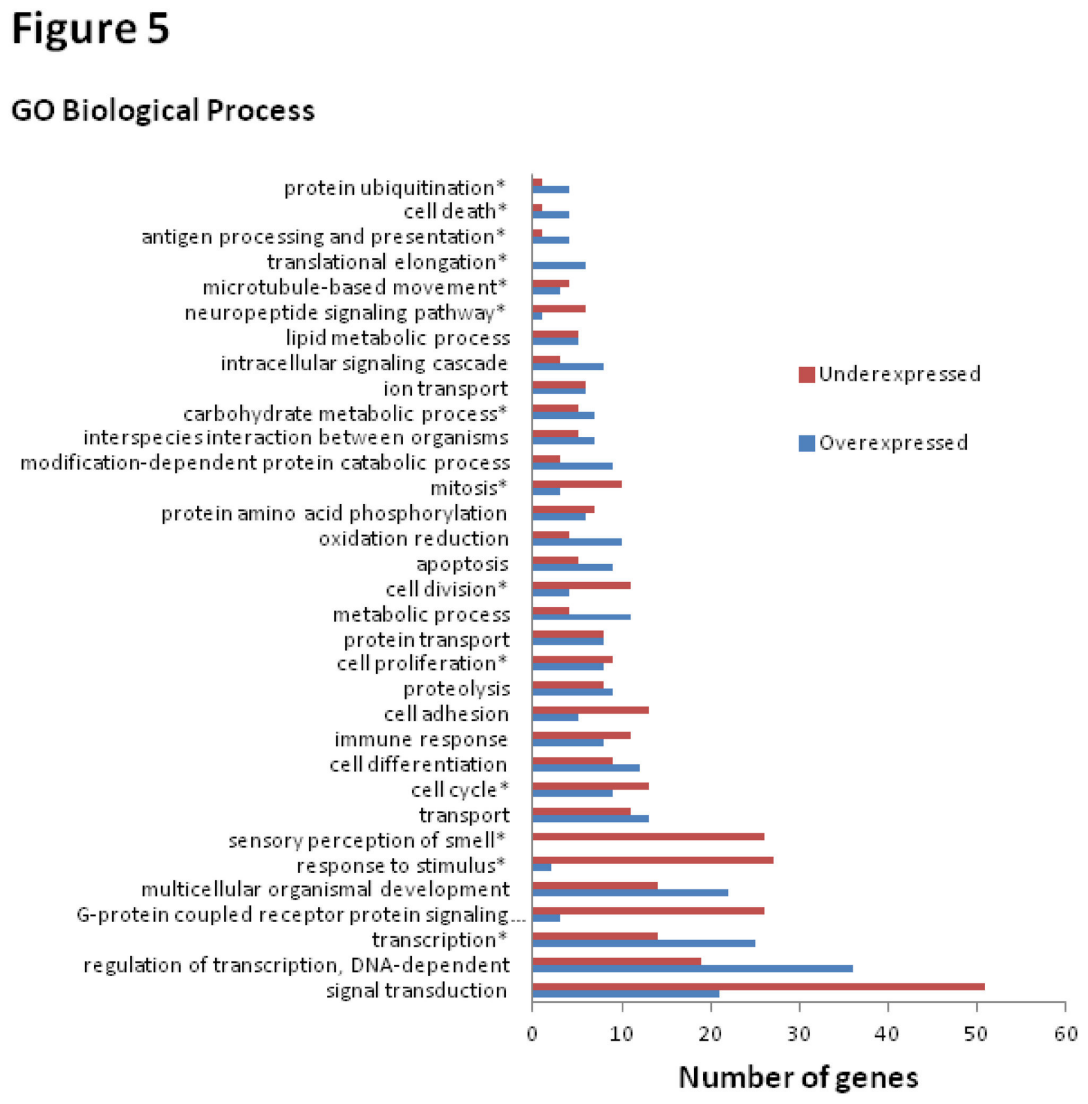
Gene Ontology (GO) categories denoting Biological Process (BP) information for differentially expressed genes. Information is derived using Onto-Express (http://vortex.cs.wayne.edu/projects.htm). Diagram shows the number of under- and over-expressed genes for GO BP. The BP marked with stars denotes a statistically significant group (*p*<0.05). Only the groups with a gene number ≥ 5 are shown.

A more in-depth analysis of the DE genes was done using Onto-Express, which also provides graphical tree view for GO terms, with the ability to browse functional profiles of the GO tree at different abstraction levels. Results in Figures 4 and 5 represent the identified GO MF and GO BP terms amongst the DE genes, along with the number of under- and over-expressed genes for each identified term using Onto-Express. In the final GO annotation analysis, we combined the results from DAVID with Onto-Express to derive the overrepresented GO categories. Among the GO MFs of interest are the “cadmium ion binding”, “metal ion binding, “zinc ion binding”, and “calcium ion binding” genes. The GO BP categories of interest include “cell division”, “cell proliferation”, and “cell cycle”. We further evaluated these GO categories. Table 4 shows the four GO MFs associated with the metal/cadmium response, and the list of genes in each of the categories; and Table 5 shows the three GO BPs linked to cell growth and the respective genes in each category.

**Table 4:** GO Molecular Function: Metal ions.

**GO ID**	**Description**	**No of genes**	**Gene members**
GO:0046870	cadmium ion binding	5	**MT1X**, **MT1M**, **MT1H**, **MT1G**, **MT1F**
GO:0008270	zinc ion binding	71	**ZNF552**, **CYHR1**, **ZNF90**, RACGAP1, C20orf12, RNF183, **PDLIM1**, **RNF4**, **MT2A**, **MT1X**, **MT1M**, **MT1H**, **MT1G**, **MT1F**, **RING1**, **RNF135**, CPN1, **RNF8**, **RASA3**, **RABGGTA**, **MMP17**, **ZNF467**, ZNF831, ADAM5P, **ADAM15**, FBLIM1, **PRKCZ**, ZNF256, **RFFL**, **RASGRP1**, AGAP7, **CBFA2T3**, **LASP1**, **SNAI3**, **LMCD1**, AGAP4, FAM90A7, CA4, **ZNF282**, **ZNHIT2**, **ZFP36L2**, **FBXO43**, CA14, ZNF404, **MEX3D**, **ZNF286A**, **JUB**, RNF39, **SOLH**, SYTL5, TRIM17, ZDHHC9, ZIM3, DPF3, EGR1, **P2RX4**, **RUFY1**, **ZMIZ1**, CIT, **STAC2**, **ZNF709**, TRIM49, CPO, **ZNF296**, **RNF208**, ATXN7, **ZXDA**, **RREB1**, ZFAND2A, **ZNF554**, **ZNF187**
GO:0005509	calcium ion binding	31	AVIL, **MYL12A**, CRP, **SYT7**, VSNL1, **MMP17**, LRP2, CADPS, **RASGRP1**, **SLC25A24**, **PLS3**, PLA2G2A, CIB3, F8, **FKBP9**, **FAHD1**, **ATP2A3**, EMR1, **ASGR1**, OCM, **SGSH**, **ANXA3**, **ANXA2P2**, **ANXA2**, AMY2A, EMR3, **S100A10**, **CD209**, FSTL5, **DAG**1, CADPS2
GO:0005506	iron ion binding	2	**OGFOD1**,NDUFS7

Genes that are highlighted in bold are over-expressed, and the rest of the genes are under expressed.

**Table 5:** GO Biological Process: Cell Growth.

**GO ID**	**Description**	**No genes**	**Gene members**
GO:0051301	cell division	15	**CCNE2**, **RNF8**, WEE1, CKS1B, SETD8, CENPF, CCNF, **CCNE1**, CCNB1, **SAC3D1**, NCAPD2, BUB1, OIP5, NUSAP1, CIT
GO:0007049	cell cycle	22	RACGAP1, **CCNE2**, **PKMYT1**, **RNF8**, WEE1, KAT2B, CKS1B, SETD8, **CLSPN**, **MAPK6**, CCNF, **CCNE1**, CCNB1, **SAC3D1**, NCAPD2, BUB1, OIP5, NUSAP1, **JUB**, CIT, **PCNP**, PSRC1
GO:0008283	cell proliferation	17	MPL, CKS1B, **CREG1**, CENPF, PRG4, LRP2, THPO, **CBFA2T3**, BUB1, **BST2**, **ZFP36L2**, **ERF**, **BIN1**, AMELX, **SLC29A2**, NANOG, **DAB2**

Genes that are highlighted in bold are over-expressed, and the rest of the genes are under expressed.

### Confirmation of DE genes using quantitative RT-PCR

**Figure 6:**
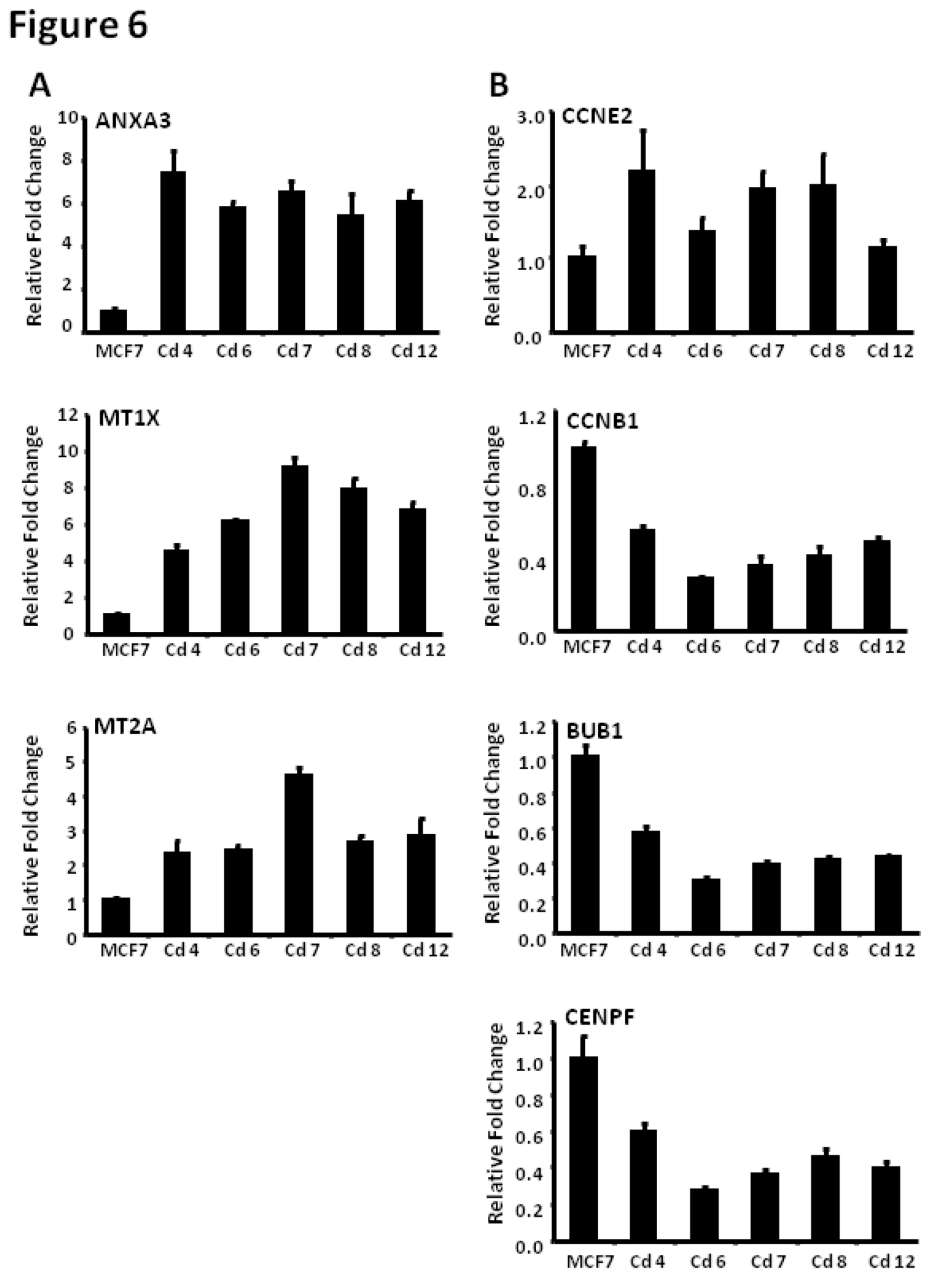
Confirmation of DE genes using quantitative RT-PCR. MCF-7 and MCF-7-Cd clonal cell lines (Cd-4, -6, -7, -8, and -12) were plated in 10 cm plates and total RNA was isolated for gene expression analysis using quantitative RT-PCR. Data are presented as relative fold changes with MCF-7 as the control and all fold changes are normalized to GAPDH (relative fold=2^∆∆Ct gene/∆∆CtGAPDH^) with p<0.001.

Differential expression of some of the genes identified in Tables 4 and 5 was further verified using quantitative RT-PCR. Total RNA from both parental MCF-7 and cadmium-exposed cell lines was isolated for gene expression analysis using gene specific primers and SYBR-green based quantitative-PCR. Relative fold changes were calculated based on expression levels in parental MCF-7 cells and subsequently normalized to the housekeeping gene, GAPDH (glyceraldehyde dehydrogenase). Consistent with Table 4, results in Figure 6A confirm that the expressions of ANXA3, MT1X and MT2A were elevated in cells chronically exposed to cadmium, with fold changes ranging from 2- to 10-fold (p < 0.001). Figure 6B shows the fold changes of cell cycle related genes— CCNE2, CCNB1, BUB1 and CENPF (p < 0.001). Among these genes, only CCNE2 is up-regulated in cells chronically exposed to cadmium, whereas CCNB1, BUB1 and CENPF are all down-regulated. Specific fold changes are summarized in Table 6 for each of the cadmium cell lines.

**Table 6:** Summary of Gene Expression Fold Changes.

	**MCF7**	**Cd4**	**Cd6**	**Cd7**	**Cd8**	**Cd12**
**ANXA3**	1.00 ±0.12	7.38 ±1.09	5.77 ±0.35	6.54 ±0.47	5.44 ±0.99	6.05±0.056
**MT1X**	1.00 ±0.08	4.49 ±0.34	6.13± 0.082	9.10 ±0.51	7.89± 0.62	6.77 ±0.41
**MT2A**	1.00 ±0.05	2.37 ±0.32	2.42 ±0.17	4.58 ±0.23	2.64 ±0.21	2.88 ±0.49
**CCNE2**	1.00 ±0.16	2.18 ±0.56	1.35 ±0.19	1.95 ±0.23	1.99 ±0.43	1.14± 0.10
**CCNB1**	1.00 ±0.04	0.55 ±0.02	0.28 ±0.01	0.35 ±0.05	0.41 ±0.04	0.49 ±0.03
**BUB1**	1.00 ±0.07	0.57 ±0.04	0.30 ±0.02	0.39 ±0.02	0.41 ±0.02	0.43 ±0.01
**CENPF**	1.00 ±0.12	0.60 ±0.04	0.27 ±0.02	0.36 ±0.03	0.46 ±0.04	0.39 ±0.04

White: Genes over-expressed in cadmium exposed cells; Gray: Genes down-regulated in cadmium exposed cells.

## Discussion

Multiple studies have suggested that cadmium functions as a metalloestrogen, mimicking the actions of estrogen to promote breast cancer cell proliferation via the activation of the estrogen receptor (ERα) [16,18,19]. Additionally, studies have also demonstrated that exposure to cadmium is associated with the development of more malignant tumors [6,24,25]. While acute cadmium exposure and correlation studies exist at the tumor tissue level, there are very few cellular models aimed at studying chronic cadmium exposure and breast cancer. We have previously developed clonal cadmium cell lines (MCF-7-Cd4, -Cd6, -Cd7, -Cd8 and -Cd12), derived from cells exposed to cadmium for over 6 months, which serve as a cell culture model to study chronic cadmium exposure [20]. While we have previously demonstrated that prolonged exposure to cadmium induces cellular changes [20]— most likely due to changes in gene expression— the impacts of chronic cadmium exposure on global gene expression remains unclear. To establish expression profiles of cells chronically exposed to cadmium, we used microarray analysis to investigate the effects of prolonged cadmium exposure on gene expression in comparison with control MCF-7 breast cancer cells. High throughput microarray technology is commonly used to identify differentially expressed genes, and previous research using microarray platforms has demonstrated its potential in case-controlled gene expression comparisons [39,40]. Hierarchical clustering analysis of the microarray data (Figure 1) confirmed that distinct expression patterns exist between parental MCF-7 and cadmium-adapted cells, thus warranting further analysis.

Results in Tables 2 and 3 provide an abbreviated lists of over- and under-expressed genes in cells chronically exposed to cadmium. Genes with known associations to breast cancer were identified using Genes-to-Systems Breast Cancer Database— G2SBC (http://www.itb.cnr.it/breastcancer/), which contains information about genes that have been previously reported in the literature to be associated with breast cancer. Furthermore, Gene Expression Atlas (GXA) from EMBL-EBI database [34] was used to further investigate DE genes by searching for previously described gene expression patterns associated with breast cancer and different biological/experimental conditions, such as estrogen treatment. Genes associated with breast cancer were then organized and represented in a heat map (Figure 2), and similar to the hierarchical clustering in Figure 1, there is a clear expression pattern among the breast cancer associated genes in cadmium-exposed samples. Interestingly, among the over-expressed genes (Table 2), MT2A (metallothionein 2A) and CCNE1 (cyclin E1) are associated not only with breast cancer, but also with cadmium induction [19,41-43] suggesting that the microarray analysis is identifying genes relevant to both cadmium exposure and breast cancer.

More in-depth analysis was accomplished using functional annotations; and an initial overview of the functional annotation of DE genes in terms of molecular functions and biological processes was obtained by using DAVID (Figure 3). Since Gene Ontology (GO) is the *de facto* method for functional annotation, tools that primarily use GO as their annotation resource were initially used in gene enrichment analysis. As there is no standard to determine which functional enrichment analysis tools should be used, it is advantageous to run more than one tool in order to compensate for any weaknesses. DAVID was used for an initial analysis to identify the most significantly enriched terms, but because of high stringency, there is a risk for neglecting some weakly enriched terms, which may bias the biological conclusion. Therefore, we also performed a functional annotation analysis using Onto-Express (Figure 4 and 5) to give us a broader picture of cadmium’s effect. The results from DAVID complemented the results from Onto-Express which allow customized analysis with the possibility of collapsing or expanding GO term nodes (and calculate corresponding *p*-values) to give us more in-depth analysis of the DE genes. 

From this analysis, we identified GO categories of interest, including genes that are associated with cadmium and other divalent metal ions (i.e. zinc and calcium). There were four GO terms identified in this category: cadmium-, zinc-, calcium- and iron-ion binding genes (Table 4). As expected, many of metallothionein genes that are known to be induced by zinc (MTA2, MT1X, etc.) and play important roles in the detoxification and transport of metal ions [44-46], were also induced by chronic cadmium exposure. Consistent with this observation, a previous study carried out on both immortalized human breast epithelial cells (HB2) and ER-negative breast cancer cells (MDA-MB-231) also reported the induction of multiple metallothionien genes (MT1F, MT1L, etc.) in response to cadmium exposure [47]. Furthermore, cadmium has been shown to induce the expression of metallothionein genes in other cancer cell types including prostate, testes, liver and lymphocytes [48-50]. Other metal binding genes that were elevated in response to chronic cadmium exposure included MMP17 (matrix metalloproteinase-17), ANXA3 (annexin A3) and STAC2 (Src homology 3 and cysteine-rich domain-containing protein 2). The ability of cadmium to stimulate the expression of metal-binding proteins is likely associated with its ability to mimic divalent cations like calcium [51,52] and/or replace cations like zinc [53-57]. Specifically, cadmium has been shown to replace the zinc ions within the DNA binding domain of the estrogen receptor [58,59]. 

The second GO category that underwent further analysis involved cell growth, which included three GO terms: cell cycle, cell division and cell proliferation (Table 5). Previous studies have implicated cadmium in mediating breast cancer cell proliferation [19,60,61], and in line with these previous reports, our microarray analysis identified over 35 growth-related genes deregulated in cells chronically exposed to cadmium. Among these are CCNE1 (cyclin E1), CCNE2 (cyclin E2), CCNB1 (cyclin B1), and CCNF (cyclin F), all of which are direct regulators of the cell cycle. Cyclin E1 and 2— known to be involved in the regulation of the G1/S transition— are increased in cells chronically exposed to cadmium. Induction of cyclin E1 was also identified in our previous acute cadmium study on breast cancer cells [19]. On the other hand, cyclin B1 and F important for regulating the G2/M transition [62-68] are both down-regulated in the cadmium-adapted cells. The deregulation of any of these cyclins has been shown to be sufficient to promote cancer cell proliferation [41,67-73]. Furthermore, in addition to the cyclins, two other proteins that regulate the cell cycle— wee1 and BUB1— were also down-regulated. Wee1 is a serine/threonine protein kinase that catalyzes the inhibitory tyrosine phosphorylation of cdk1/cyclin B complex and coordinates the transition between DNA replication and mitosis, whereas BUB1 is a mitotic checkpoint serine/threonine-protein kinase. 

Expression of a select number of genes from both GO categories (cadmium/metal binding and cell growth) were further evaluated using quantitative RT-PCR, and results confirmed that the specific genes were in fact deregulated in cells chronically exposed to cadmium in comparison to parental MCF-7 cells (Figure 6). While the qRT-PCR data were consistent with microarray data regarding the direction of change (up- or down-regulated), the relative fold changes found with qRT-PCR were much greater than those calculated in the microarray analysis. This is likely due to the higher noise:signal ratio associated with microarray analyses [74]. 

While this study was focused on ER-positive breast cancer cells, chronic cadmium exposure is also relevant to other cancer cell types. As noted earlier, some of the genes identified in this study were also deregulated in other cancer cells when exposed to cadmium [47-50]. A more recent study by Garrett and colleagues on acute (1 day) and chronic (13 days) cadmium exposure in kidney tubule cells also used microarray analysis to examine the gene expression profiles of cells exposed to cadmium [75]. Contrary to our study, the genes most significantly altered by chronic cadmium exposure did not include many metallothionein genes. However, the authors did identify LASP1 (LIM and SH3 protein 1) and ANXA2P2 (annexin A2 pseudogene 2) as being elevated and FAM182A (family with sequence homology 182) as being down-regulated, which is consistent with our results. We speculate that the observed differences between Garrett’s study and ours are likely associated with several variable factors including cell type (kidney tubule cells versus breast cancer cells), cadmium concentration (10^-5^ M versus 10^-7^ M), and length of cadmium exposure (13 versus >180 days). Unfortunately, the authors only reported a short list of genes with p<0.001, even though a much larger set of genes with p<0.01 was apparently generated, and it would be interesting to compare our results with this larger data set. 

It is important to note that our study has only identified genes that were deregulated in all five clonal cadmium cells lines. As a result, there may be genes deregulated in *only* a subset of the cadmium clones that were not picked up in this study. Of particular interest is SDF-1, a gene that we have previously shown to be elevated in over 70% of the cadmium-adapted clonal cell lines generated [20]. Consistent with this, our microarray data also identified SDF-1 as one of the genes elevated in four of the five MCF-7-Cd cell lines used in this study (data not shown). Therefore, future analysis using less stringent criteria may identify additional genes relevant to cadmium-induced carcinogenesis.

This study is the first to identify gene expression patterns that differentiate breast cancer cells exposed to cadmium for prolonged periods of time versus those that have not. Our results demonstrate that prolonged cadmium exposure directly results in the deregulation of genes previously identified as being associated with breast cancer. While some of the deregulated genes were expected (cadmium/metal response genes), many are newly identified as cadmium-associated genes. Furthermore, these cadmium-associated genes are involved in multiple biological processes (i.e. cell growth and apoptosis) and molecular functions (i.e. cadmium/metal ion binding and transcription factor activity). The most intriguing finding from this study is that all five clonal cadmium cell lines share a common gene expression signature, suggesting that mammary tumors with high cadmium levels may share a similar expression profile (Figures 1 and 2). Therefore, future microarray analysis of mammary tumors with elevated cadmium levels will provide insight into the potential role of the bio-accumulated cadmium in breast cancer development and progression. Our results demonstrate the cellular and molecular impacts of cadmium on breast cancer carcinogenesis and emphasize the importance of studying the effects of chronic cadmium exposure on breast cancer.

## Supporting Information

Table S1 **Complete list of differentially expressed genes.**
(XLS)
