# Correction: Using Expression Profiling to Understand the Effects of Chronic Cadmium Exposure on MCF-7 Breast Cancer Cells

**DOI:** 10.1371/annotation/4737b7e4-0d5e-4f86-b916-bda9b6cabd84

**Published:** 2014-01-16

**Authors:** Zelmina Lubovac-Pilav, Daniel M. Borràs, Esmeralda Ponce, Maggie C. Louie

This article was republished on January 15th, 2014 due to data that was erroneously included in the Supporting Information section.

